# Assessing the healthcare quality issues for digital incident reporting in Sweden: Incident reports analysis

**DOI:** 10.1177/20552076231174307

**Published:** 2023-05-08

**Authors:** Md Shafiqur Rahman Jabin, Mary Steen, Dianne Wepa, Patrick Bergman

**Affiliations:** 1 Department of Medicine and Optometry, 4180 Linnaeus University, Kalmar, Sweden; 2 Faculty of Health Studies, 1905 University of Bradford, Bradford, UK; 3 Department of Nursing, Midwifery and Health, 5995 Northumbria University, Newcastle upon Tyne, UK

**Keywords:** Patient safety, quality improvement, software issues, hardware issues, health information technology, human factors, technical factors, classification system, training and education

## Abstract

**Objective:**

This study explored healthcare quality issues affecting the reporting and investigation levels of digital incident reporting systems.

**Methods:**

A total of 38 health information technology-related incident reports (free-text narratives) were collected from one of Sweden's national incident reporting repositories. The incidents were analysed using an existing framework, i.e., the Health Information Technology Classification System, to identify the types of issues and consequences. The framework was applied in two fields, ‘event description’ by the reporters and ‘manufacturer's measures’, to assess the quality of reporting incidents by the reporters. Additionally, the contributing factors, i.e., either human or technical factors for both fields, were identified to evaluate the quality of the reported incidents.

**Results:**

Five types of issues were identified and changes made between before-and-after investigations: Machine to software-related issues (*n*  =  8), machine to use-related issues (*n*  =  5), software to software-related issues (*n*  =  5), use to software-related issues (*n*  =  4) and use to use-related issues (*n*  =  1). Over two-thirds (*n*  =  15) of the incidents demonstrated a change in the contributing factors after the investigation. Only four incidents were identified as altering the consequences after the investigation.

**Conclusion:**

This study shed some light on the issues of incident reporting and the gap between the reporting and investigation levels. Facilitating sufficient staff training sessions, agreeing on common terms for health information technology systems, refining the existing classifications systems, enforcing mini-root cause analysis, and ensuring unit-based local reporting and standard national reporting may help bridge the gap between reporting and investigation levels in digital incident reporting.

## Introduction

The functions of medical incident reporting systems are to collect information concerning incidents from healthcare professionals and to investigate and analyse any problems/issues identified. This approach then enables the development of preventive strategies and assists in identifying priorities (locally, regionally, and nationally), and thus provides opportunities for valuable feedback (evidence of actions).^1^ Medical incident reporting systems are usually digital, voluntary, non-punitive, and craft- (or speciality-) based reporting systems, mainly designed to provide customised feedback for healthcare quality and patient safety improvement.^1,2^ Despite many advantages, several drawbacks in medical incident reporting systems can be outlined. Such drawbacks may include: Unsuitability for elucidating complex problems,^3^ bias in the reports regarding reporting sites, modality, and other parameters,^4^ limited in-depth systematic analyses, and inadequate value gained compared to the time taken for a report in busy and complex healthcare practice.^4^

One of the challenges of Swedish incident reporting systems is that these are currently decentralised. Even though each Swedish region has established computerised reporting systems to which any healthcare professionals can submit incident reports, each region has its own distinct online reporting system. These varying systems include LISA (Kalmar), Synergi (Uppsala, Jonköping), and Platina (Halland, Gävleborg), and this diversity creates difficulties in interoperability and cross-regional comparisons, thereby hindering the ability to learn from mistakes. Moreover, these reporting systems have different user groups and role positions comprising the basic level as the reporting level and, subsequently, the managing and investigation levels.^5^

A new stipulation by the National Board of Health and Welfare has been set to regulate and manage incidents with medical devices to address the issues concerning healthcare quality and patient safety.^6^ In Sweden, once a healthcare professional reports an incident about a medical device or health information technology (HIT) system, the manufacturer must investigate the reports and identify the necessary measures to minimise the risks. During the investigation of the reports, the manufacturer can request additional information from a designated healthcare organisation in which an incident occurred. The manufacturer, in return, must report to the Medical Products Agency (MPA) after they have recognised a (potential) causal link between an incident and the HIT systems.^6^

Various types of issues may arise concerning the incidents associated with HIT systems. Some problems are technical enfolding software or hardware design issues. Other human or organisational issues related to sociotechnical contexts influence the human–computer interface.^7^ HIT system software issues can be of different types, such as systems configuration (problems with default settings), software accessibility and availability,^8^ and software functionality (poor user interfaces, fragmented displays).^9^ These problems can range from inconvenience and workflow interruptions to patient harm and affect whole systems and multiple facilities.^10^

These HIT problems, their contributing factors, and their impact on patients, healthcare professionals, and healthcare organisations can be determined using different qualitative approaches, such as inductive and deductive approaches. The inductive approach may involve content or thematic analyses, whereas the deductive method may use an existing framework or theoretical model, for example, the human–computer interaction model^11^ and HIT classification system (HIT-CS).^10^ The HIT-CS is a ‘bottom-up’ approach for identifying HIT-related issues developed by Magrabi et al.^10^ A single incident can be classified into more than one incident type or type of issue using this classification system. The existing framework also provides the ability to identify the factors underpinning the incidents and the consequences of the incidents that occurred.^10^

So far, a limited amount of research has been conducted to assess the quality of reported incidents, particularly in the context of HIT-related incidents. Several studies have evaluated the quality improvement of the incident reporting systems,^12^ quality costs within electronic incident reporting and recording systems,^13^ the impact of critical incident reporting and its improvement in neonatal practice^14^ and intensive care unit.^15^ However, it appears that no research has been conducted to assess the quality of the incident reports associated with HIT and to identify the differences in narrative texts between before-and-after investigations. Therefore, there is an identified need for a qualitative evaluation of the HIT-related incidents between the reporter's event description and the manufacturer's conclusion after investigation. An existing framework, such as HIT-CS, has been identified as helpful for analysing HIT-related incidents to determine the dissimilarities in the HIT-incident reports between the reporting and investigation levels.

This study aims to explore HIT-related incidents with the help of the HIT-CS to assess the quality of the incident narration by the reporters. The study examines how the incident narration and the conclusion changes after the investigation was conducted by the manufacturers. Overall, it investigates the healthcare quality issues affecting the reporting and investigation levels of the incident reporting systems.

## Methods

### Data collection

The information about what went wrong with HIT systems was collected from one of the national incident reporting repositories in Sweden. The organisation responsible for incident repositories has adopted the initiative for implementing incident reporting by the healthcare professionals through their respective county councils’ digital incident reporting systems to improve healthcare quality, patient safety, and throughput. A total of 38 HIT-related incident reports were delivered, from various regions of Sweden (de-identified) from June 2019 to June 2021 (2 years). All of these incidents were selected based on the fact that they underwent complete investigation by the product manufacturers and the data provider organisation, and that they were isolated (before delivery) from the general set of medical device-related incidents.

These reports were free-text narratives, containing three main fields: event description (by the reporter), manufacturer's conclusion, and manufacturer's measures (action taken). There were other inconsistent information fields, such as the number of affected patients and the number of affected medical systems. A member of the data provider partially edited the information fields to deidentify the identifying, sensitive, and personal information. The incident reports were delivered in Swedish and then translated into English by a linguistic expert with proficiency in both languages.

The principal investigator made an attempt to communicate and follow up with the data provider to gain a deeper insight and understanding of things that went wrong and to extract additional information relating to the context. Further information was required to explore any regularly used recovery strategies and how incidents might have been prevented or minimised. However, no response was received, perhaps due to a major administrative change that occurred in the data provider's organisation as a result of the ongoing pandemic.

### Data analysis

A preliminary check was performed to ensure if the delivered incidents were HIT-related incidents or not. A HIT algorithm^16^ was used for identifying HIT-related incidents. An incident was included if it contained a HIT system but was not a part of medical equipment or an implantable device.

The included incidents were analysed using an existing framework proposed by Magrabi et al.,^10^ i.e., the HIT-CS. The HIT-CS helps identify the type of issues and their consequences from the incident reports consisting of several structured and free text fields. With the help of this existing framework, incidents can first be categorised into use or machine-related (types of) issues and then software or hardware-related issues. The consequences were assigned using a standard approach in line with the classification system (see Table 2).

The HIT-CS were applied in both fields, ‘event description’ by the reporters (before investigation) and ‘manufacturer's measures’ (after investigation), to identify the type of issues and type of consequences. This classification helped to assess the difference in the free-text narratives of the two fields, particularly the quality of reporting incidents by the reporters. Additionally, the contributing factors, i.e., either human or technical factors for both fields, were identified to evaluate the quality of the reported incidents (event description).

Kappa score calculation was performed to ensure inter-rater reliability. Since more than one type of issue could have been identified from a single incident report, the primary incident type was considered for inter-rater reliability (kappa score calculations). Two investigators were involved in classifying the incidents independently for the reliability of the coding. When the coders disagreed on any category, the incident was re-examined, and a consensus category was allotted.

## Results

A batch of 38 incidents was delivered for analysis. Of 38 incidents, one was excluded because it could not be categorised as a HIT-related incident. Of the remaining 37 incidents, two incidents had no narrative text provided (or missing) for the ‘manufacturer's measures’ (see Table 1: IR_32,36_), and two incidents had inadequate narrative texts or ineligibility for HIT classification (see Table 1: IR_17,26_).

**Table 1.** Description of incident classification of the total sample (*n*  =  37) at reporting and investigation levels.

Incident Report No.	Event description by the reporter (Before investigation)			Manufacturer's conclusion (After investigation)		
	Type of issue	Consequences	Contributing factor	Type of issue	Consequences	Contributing factor
IR_1_	Wrong entry/retrieval and wrong output	An arrested or interrupted sequence or a near miss	HF	Interface with other software systems or components	An arrested or interrupted sequence or a near miss	TF
IR_2_	Wrong output	An arrested or interrupted sequence or a near miss	TF	Wrong output	An arrested or interrupted sequence or a near miss	TF
IR_3_	No output	Harm to a patient or an adverse event	HF	Software functionality	Harm to a patient or an adverse event	TF
IR_4_	No output	Incidents with no noticeable consequence	HF	Software functionality	Incidents with no noticeable consequence	TF
IR_5_	Wrong output and Interface with other software system or component	Incidents with no noticeable consequence	TF	Wrong output and interface with other software system or component	Harm to a patient or an adverse event	HF
IR_6_	Wrong output	An arrested or interrupted sequence or a near miss	TF	Wrong entry/retrieval	An arrested or interrupted sequence or a near miss	HF
IR_7_	Wrong output	An arrested or interrupted sequence or a near miss:	TF	Software functionality	An arrested or interrupted sequence or a near miss	TF
IR_8_	Wrong output and Record migration	An arrested or interrupted sequence or a near miss:	TF	Wrong output and Record migration	An arrested or interrupted sequence or a near miss:	TF
IR_9_	System configuration		TF	System configuration		TF
IR_10_	No output	Incidents with no noticeable consequence	TF	Wrong entry/retrieval	Harm to a patient or an adverse event	HF
IR_11_	No entry/retrieval		HF	Wrong entry/retrieval and system configuration		HF and TF
IR_12_	No output and software functionality	Incidents with a noticeable consequence but no patient harm	TF	No output and Software functionality	A hazardous event or circumstances	TF
IR_13_	Interface with other software systems or components	Incidents with a noticeable consequence but no patient harm	TF	Interface with other software systems or components	Incidents with a noticeable consequence but no patient harm	TF
IR_14_	No output	An arrested or interrupted sequence or a near miss	TF	No entry/retrieval	An arrested or interrupted sequence or a near miss	HF
IR_15_	No output	Harm to a patient or an adverse event	TF	Wrong entry/retrieval	Harm to a patient or an adverse event	HF
IR_16_	Wrong entry/retrieval	Harm to a patient or an adverse event	HF	System configuration	Harm to a patient or an adverse event	TF
IR_17_	System configuration		TF			
IR_18_	Interface with other software systems or components	An arrested or interrupted sequence or a near miss	TF	Interface with other software systems or components	An arrested or interrupted sequence or a near miss	TF
IR_19_	Data storage and backup	Incidents with no noticeable consequence	TF	Data storage and backup	Incidents with no noticeable consequence	TF
IR_20_	Software not accessible	Incidents with no noticeable consequence	TF	Software not accessible	Incidents with no noticeable consequence	TF
IR_21_	No output	Harm to a patient or an adverse event	TF	System configuration	Harm to a patient or an adverse event	TF
IR_22_	No output	Incidents with a noticeable consequence but no patient harm	TF	System configuration	Incidents with a noticeable consequence but no patient harm	TF
IR_23_	Software functionality	Incidents with a noticeable consequence but no patient harm	TF	System configuration	Incidents with a noticeable consequence but no patient harm	HF
IR_24_	System configuration	Incidents with no noticeable consequence	HF	System configuration	Incidents with no noticeable consequence	TF
IR_25_	Interface with other software systems or components	Harm to a patient or an adverse event	TF	Interface with other software systems or components	Harm to a patient or an adverse event	TF
IR_26_	Software not accessible	Harm to a patient or an adverse event	TF			
IR_27_	Software not available or not licensed	Incidents with a noticeable consequence but no patient harm	TF	Interface with other software systems or components	Incidents with a noticeable consequence but no patient harm	TF
IR_28_	No output	Harm to a patient or an adverse event		Wrong entry or retrieval	Harm to a patient or an adverse event	HF
IR_29_	Interface with other software systems or components	Harm to a patient or an adverse event	TF	Software functionality	Harm to a patient or an adverse event	TF
IR_30_	Software functionality	a hazardous event or circumstances	TF	Interface with other software systems or components	Harm to a patient or an adverse event	TF
IR_31_	No entry/retrieval	Harm to a patient or an adverse event	TF	System configuration	Harm to a patient or an adverse event	HF
IR_32_	Software not available or not licensed	An arrested or interrupted sequence or a near miss	HF			
IR_33_	No output	Harm to a patient or an adverse event	TF	System configuration	Harm to a patient or an adverse event	TF
IR_34_	No output	Incidents with a noticeable consequence but no patient harm	HF	System configuration	Incidents with a noticeable consequence but no patient harm	TF
IR_35_	System configuration	Harm to a patient or an adverse event	TF	Software functionality	Harm to a patient or an adverse event	TF
IR_36_	Partial output	Harm to a patient or an adverse event	TF			
IR_37_	Partial output	An arrested or interrupted sequence or a near miss	TF	System configuration	An arrested or interrupted sequence or a near miss	HF

IR: incident report; HF: human factor; TF: technical factor.

Inter-rater reliability before the investigation (event description by the reporters) between the coders were: primary incident type, к  =  0.87 (*p* < 0.001, 95% CI 0.80–0.94); contributing factors, к  =  0.86 (*p* < 0.001, 95% CI 0.79–0.95); and consequences was к  =  0.82 (*p* < 0.001, 95% CI 0.74–0.92).

Inter-rater reliability after the investigation (manufacturers’ measures) between the coders were primary incident type, к  =  0.89 (*p* < 0.001, 95% CI 0·82–0.99); contributing factors, к  =  0.85 (*p* < 0.001, 95% CI 0.71–0.98); and consequences, к  =  0.87 (*p* < 0.001, 95% CI 0·75–0.99).

### Incident characteristics

The total sample of the incidents (*n*  =  37) was classified into three classes: Types of issues and consequences according to the HIT-CS and the contributing (human versus technical) factors. A comparison was drawn to illustrate the characteristics of the incidents before and after the investigation. A detailed description of the incident classification of the total sample (*n*  =  37) at the reporting and investigation levels is presented in Table 1.

#### Types of issues

According to HIT-CS, an incident can be characterised by more than one issue. It is customary for the incidents to be classified into either use or machine-related issues and then into hardware or software-related issues. However, no hardware-related issues were identified among the total sample (*n*  =  37).

Based on the reported events (before investigation) identified, 41 types of issues were present, of which four incidents were characterised by more than one issue (see Table 1: IR_1,5,8,12_). Among use-related problems, the most common was ‘wrong entry/retrieval’ (*n*  =  3), and machine-related, was the ‘wrong output’ (*n*  =  5) (see Table 2). Of software-related issues, the most frequent was the software issues related to ‘interface with other software systems or components’ (*n*  =  5) (see Table 2).

**Table 2.** Incident characteristics using the health information technologyclassification system (HIT-CS) (types of issues and consequences) and contributing (human versus) factors.

Category	Before investigation (*n*)	After investigation (*n*)
Types of issues (HIT-CS)		
**Use-related issues**		
Wrong entry/retrieval	3	5
Did not enter/retrieve	2	1
**Machine related issues**		
Wrong output	5	3
Partial output	2	0
No output	11	1
**Software issues**		
Software not available or not licensed	2	0
Software not accessible	2	1
Software functionality	3	6
System configuration	4	11
Interface with other software systems or components	5	7
Data storage and backup	1	1
Record migration	1	1
Total	41	37
Contributing (human vs technical) factors		
Technical factor	28	24
Human factor	8	10
Total	36	34
Consequences (HIT-CS)		
Harm to a patient or an adverse event	12	13
An arrested or interrupted sequence or a near miss	9	8
Incidents with a noticeable consequence but no patient harm	6	4
Incidents with no noticeable consequence	6	5
A hazardous event or circumstances	1	1
Total	34	31

After the investigation, the manufacturer's conclusion identified 37 HIT issues, of which four were grouped into more than one issue (see Table 1: IR_5,8,11,12_), and four incidents could not be categorised into any issues due to no or inadequate information (see Table 1: IR_17,26,32,36_). Among use-related problems, the most common was ‘wrong entry/retrieval’ (*n*  =  5); and machine-related, was the ‘wrong output’ (*n*  =  3) (see Table 2). Among software-related issues, the most frequent was the software issues related to ‘system configuration’ (*n*  =  11) (see Table 2).

#### Human versus technical factors

The incidents were further classified to check which contributing factors were involved, either human or technical factors. Before the investigation, 36 contributing factors were identified (one for each incident), except one with inadequate details (see Table 1: IR_28_). Over three-quarters, (*n*  =  28) of incidents were contributed by technical factors, and the rest (*n*  =  8) by human factors (see Table 2).

The narrative texts by the manufacturer's conclusion determined 34 contributing factors, of which one incident was caused by two (human and technical) factors (see Table 1: IR_11_). No contributing factor could be identified due to insufficient details in the report (see Table 1: IR_17,26,32,36_). Of 34 factors: 24 were technical, and the rest were human factors (*n*  =  10) (see Table 2).

#### Consequences

Consequences were classified using the HIT-CS. Of 37 incidents, 34 consequences (one consequence per incident) were determined before the investigation. Three incidents could not be categorised into any consequences due to the limited information (see Table 1: IR_9,11,17_). Of 34 identified consequences, over one-third (*n*  =  12) resulted in harm to a patient or an adverse event, and over one-quarter led to ‘an arrested or interrupted sequence or a near miss’ (*n*  =  9) (see Table 2).

After the investigation, 31 consequences were identified from the manufacturer's conclusion. Due to the lack of information, no consequence could be identified for six incidents (see Table 1: IR_9,11,17,26,32,36_). Of these consequences, over one-third (*n*  =  13) caused ‘harm to a patient or an adverse event’, and over one-quarter resulted in ‘an arrested or interrupted sequence or a near miss’ (*n*  =  8) (see Table 2).

### Comparison of the incident classification (before and after investigation)

Of the total sample (*n*  =  37), seven incidents had no alterations in the classification (after investigation) for any classes: Types of issues, consequences, and contributing factors (see Table 1: IR_2_ and IR_13_). Only one incident had changed (after investigation) for all three classes: types of issues, consequences, and contributing factors (see Table 1: IR_10_). The remaining incidents indicated variations in the narrative texts (after investigation) either for one or two classes.

#### Types of issues

Of 37 incidents, over half (*n*  =  22) of the incidents identified types of issues (see Table 3) which were different between the narrative texts before the investigation (by the reporter) and after the investigation (by the manufacturer).

**Table 3.** Description of changes in the incident classification (types of issues) from reporting to investigation level.

Incident report (IR) No.	Before investigation	After investigation
	**Machine-related issues**	**Software-related issues**
IR_3_	No output	Software functionality
IR_4_	No output	Software functionality
IR_7_	Wrong output	Software functionality
IR_21_	No output	System configuration
IR_22_	No output	System configuration
IR_33_	No output	System configuration
IR_34_	No output	System configuration
IR_37_	Partial output	System configuration
	**Machine-related issues**	**Use-related issues**
IR_6_	Wrong output	Wrong entry/retrieval
IR_10_	No output	Wrong entry/retrieval
IR_14_	No output	Did not enter/retrieve
IR_15_	No output	Wrong entry/retrieval
IR_28_	No output	Wrong entry/retrieval
	**Software-related issues**	**Software-related issues**
IR_23_	Software functionality	System configuration
IR_27_	Software not available or not licensed	Interface with other software systems or components
IR_29_	Interface with other software systems or components	Software functionality
IR_30_	Software functionality	Interface with other software systems or components
IR_35_	System configuration	Software functionality
	**Use-related issues**	**Software-related issues**
IR_1_	Wrong entry/retrieval	Interface with other software systems or components
IR_16_	Wrong entry/retrieval	System configuration
IR_11_	Did not enter/retrieve	System configuration
IR_31_	Did not enter/retrieve	System configuration
	**Use-related issues**	**Use-related issues**
IR_11_	Did not enter/retrieve	Wrong entry/retrieval

Five types of alterations between before-and-after investigations were identified: Machine to software-related issues (*n*  =  8), machine to use-related issues (*n*  =  5), software to software-related issues (*n*  =  5), use to software-related issues (*n*  =  4), and use to use-related issues (*n*  =  1). System configuration (*n*  =  9) and software functionality (*n*  =  5) were the most common types of issues altered after the investigation (see Table 3).

#### Human versus technical factors

Over two-thirds (*n*  =  15) of the incidents demonstrated a change in the contributing factors after the investigation. Of these, over half (*n*  =  8) altered from technical to human factors, and the rest (*n*  =  7) amended from human to technical factors (see Table 4).

**Table 4.** Description of changes in the contributing (human versus technical) factors from reporting to investigation level.

IR No.	Before investigation	After investigation
IR_1_	HF	TF
IR_3_	HF	TF
IR_4_	HF	TF
IR_5_	TF	HF
IR_6_	TF	HF
IR_10_	TF	HF
IR_11_	HF	HF & TF
IR_14_	TF	HF
IR_15_	TF	HF
IR_16_	HF	TF
IR_17_	TF	-
IR_23_	TF	HF
IR_24_	HF	TF
IR_26_	TF	-
IR_28_		HF
IR_31_	TF	HF
IR_32_	HF	-
IR_34_	HF	TF
IR_36_	TF	-
IR_37_	TF	HF

IR: incident report; HF: human factor; TF: technical factor.

#### Consequences

Only four incidents (IR_5,10,12,30_) were identified as altering the consequences after the investigation, of which three changed to harm to a patient or an adverse event. Of these three incidents, a major difference was identified for the two incidents (see Table 5); for example, incidents with no noticeable consequences resulted in harm to a patient or adverse event.

**Table 5.** Description of changes in the incident classification (consequences) from reporting to investigation level.

Incident report (IR) No.	Before investigation	After investigation
IR_5_	Incidents with no noticeable consequence	Harm to a patient or an adverse event
IR_10_	Incidents with no noticeable consequence	Harm to a patient or an adverse event
IR_12_	Incidents with a noticeable consequence but no patient harm	A hazardous event or circumstances
IR_30_	a hazardous event or circumstances	Harm to a patient or an adverse event

## Discussion

The medical incident reporting system is now an integral and essential part of modern healthcare. Managing incidents related to HIT systems is the most emerging necessity due to the rapid adoption and transformation of digital healthcare across the country. With the convenience and achievement of Swedish healthcare's prominent digital goals in every sphere, incident reporting is complementary to a more in-depth quantitative approach. Thus, an incident captured through incident reporting has the potential to characterise the problems and develop preventive and corrective strategies to mitigate the risks to patient safety. Other approaches with the conventional prospective study design would not be affordable and convenient.^1^

However, digital incident reporting is complex and dynamic and suffers from deficiencies and failures, as documented in Sweden.^5^ In this study, the incident reports before the investigation were limited by the content knowledge of the reporters. Other possibilities could be subjective impressions, sub-stranded personal bias, or lack of willingness to report diligently. Reporters, as a whole, displayed a lack of knowledge and competencies of and interest in HIT systems and their problems. Moreover, the quality of the data was varied; for example, some fields were left completely empty, or some details were entirely ignored. These hindrances added to the limitation in devising local preventive and corrective strategies.

For this study, we examined how the narrative texts or the context of the incidents change after thorough investigations of the reports by the analysts. This was performed by analysing the incidents using the HIT-CS, applied to both fields ‘event description’ by the reporter (before investigation) and ‘manufacturer’s measures’ (after investigation) to identify changes in the narration of the reports. This exploration was helpful because it would orientate the reporters and analysts to provide some context about the challenges of reporting HIT-related incidents to incident reporting systems. Thus, this study paved the way for devising a set of recommendations that need to be adopted to minimise the gap between incident narration by the reporters and the analysts.

### Types of issues

For the total sample of HIT incidents (*n*  =  37), the overall number of issues identified before and after investigations was similar (*n*  =  41 and 37, respectively). However, most of the incidents were use or machine-related issues before investigation, except for five incidents identified as software-related problems. On the contrary, the same incidents were categorised as software-related issues, except for six, which were determined as use-related incidents.

Around 59% (*n*  =  22) of the total sample demonstrated a change in ‘types of issues’ in the incident narration after the investigation. When an alteration score was provided to the incident classification for the ‘types of issues’, four (18%) of the 22 incidents had severe alteration, 12 (55%) were of moderate change, and the rest (*n*  =  6; 27%) displayed minor change after the investigation (see Table 6).

**Table 6.** Description of alteration score for the types of issues.

IR No.	Before investigation	After investigation	Alteration score
IR_1,16,11,31_	Use-related issues	Software-related issues	3
IR_6,10,14,15,28_	Machine-related issues	Use-related issues	2
IR_4,7,21,22,33,34,37_	Machine-related issues	Software-related issues	2
IR_23,27,29,30,35_	Software-related issues	Software-related issues	1
IR_11_	Use-related issues	Use-related issues	1

IR: incident report; 3: severe; 2: moderate; 1: minor.

There were consistent signals of some particular software configuration and functionality issues, which could be amenable to prevention or correction; however, the reporter did not understand the mechanism, and thus the mitigation of the risks was not possible. For instance, the care providers thought certain information was missing in the patient record; on the contrary, the patient record system functioned as intended – information not being sent was designed to meet the requirement of ‘data protection legislation’ for privacy as a default. Another example may include the invisibility of administered doses into the administration list of a patient. The reporters could scarcely know the underlying issue; therefore, there was only plain narration in the reports. This was, in fact, a functional error detected during regression testing and corrected accordingly after investigation.

These system issues were potentially amenable to system re-design or alternative administrative arrangements; rather, they were put in the ‘too hard basket’, and the system was allowed to be continuously dysfunctional. Whilst some of those software issues could sequentially be ‘designed out’, in the meantime, IT experts or medical engineers would need a conveniently available process for detecting and applying digital solutions to those issues. The deployment of immediate backup systems at very short notice would also be highly desirable for some cases.^7,17^

The reality is that when a system is being continuously used, it is being beta-tested, which is probably inevitable. This is true for such high-stakes operations, i.e., healthcare – to test systems at a level of complexity accurately, and to minimise the risks of the vagaries of the front-end operators, would be prohibitively expensive.^17^ Therefore, it is apparent, without a doubt, that the HIT systems being introduced can and continue to fail without any prior warning. Therefore, the digital backup system and contingency procedures must be in place immediately if the continuity of the system function needs to be restored.^7,16^

### Human versus technical factors

Around three-quarters of the incidents were contributed by technical factors that were scattered amongst multiple components of the forms and functions of the HIT systems. Locally developed preventive and corrective strategies to these problems related to technical factors were not possible due to the inadequate information available in the incident reports. There was an abundance of evidence of the fact that the reporters hardly understood the underlying mechanisms behind the problems. For example, an incident was thought to be contributed by a human factor – an initial assessment was made on the ground that incorrect information (belonging to another patient) in the concluding summary assessment in the patient record was sent electronically. On the contrary, the software issue was, in fact, caused by an error due to problems with the message threading.

Human errors were inevitable and accounted for one-fourth of the problems: incorrect information being sent, failure to carry out the duty, handling errors, staff unawareness, etc. It is challenging to detect the human factors unless the reporters state their own mistakes or cognitive mechanism through which they originate are detected; also difficult to prevent as they may be unintended. The convenience of machines, devices, and systems-related problems is that they can be successively ameliorated, whereas human errors by the users will remain an inherent part of the healthcare environment. Since the human factor is always an enduring part of any complex sociotechnical system, continuous refinement of professional training and safeguards for healthcare professionals would be highly recommended. Despite being the weak link, it is the human (as medical engineers or IT experts) who can fix any novel problem arising, such as the human-device interface issue.^7^

### Consequences

Initially, incidents causing patient harm were found to be contributed by technical factors; however, the scenario changed after the investigation indicating patient harm is associated with human errors. It was evident that more harm was caused by the incidents emerging from human factors than those arising from technical factors. However, careful consideration needs to be placed on this statement since our study's total sample is very small.

A number of software-related issues that were detected after the investigation caused noticeable consequences without patient harm. These problems affected multiple patients and caused major inconveniences to patients and additional workload for the healthcare professionals since the frontline operators did not go any further to keep the workflow up-to-date.

Our results are consistent with the findings by Jabin et al. and Magrabi et al., who found issues contributed by human factors caused more deleterious effects than technical factors.^10,18^ Additionally, the software issues were more prone to triggering large-scale events that affected the care management of multiple patients.^10,18^

### Challenges in the Swedish incident reporting system

Sweden, in particular, utilises the digital incident reporting system to identify the risks related to patient safety and improve healthcare quality by mitigating those identified risks with the help of preventive and corrective strategies. A recent study by Anna Hahre on the system-wide healthcare incident reporting systems in Sweden found deficiencies, summarised into three main themes.^5^ These main themes were acceptance and the use of the system, functionality and complexity, and challenges at the management level. Like any other digital system, the incident reporting system indicated variation in acceptance and use of the system in different regions and county councils. According to the study, enormous amounts of education and training were required for the staff at both reporting and investigation levels.^5^

Inconsistency in the functions of the reporting system added more complexity; for example, there were multiple business flows that did not look the same, causing the reporters to be confused at the time of reporting. The categorisation of the incidents supplemented this problem; for instance, if the reporter chose more than one category, an additional incident for the second category had to be reported. The complexity of the systems was further amplified by the advanced features, which were not fully utilised, and to some extent, not completely understood by the healthcare staff. The time pressure in the busy healthcare environment did not allow the staff to perform additional tasks, such as selecting the second category or spending more time on the advanced features.^5^

The third theme involved the challenges regarding the allocation of the roles and the classification of the events. The roles were distributed on three levels: reporting, managing, and investigation levels. Not all user groups have access to these three levels, which made it difficult to assign the appropriate staff for the classification of the incidents. The users found the reporting level simple; however, the managing and investigation level was more complex. Therefore, the logic of the system was not considered user-friendly, which led to further staff frustration.^5^

The study of these findings is in synchrony with the results obtained in our studies, for instance, the considerable difference in the types of issues identified in the narrative texts of the incidents before investigation (by the reporter) and after investigation (manufacturer's conclusion). Several other limitations were indicative in our study; for instance, the narrative texts of the reported information are limited, and inconsistency of the narrative fields – not all incidents provided information on the number of affected patients and HIT systems. Therefore, the analysis in this study had to depend on what was proffered instead of what might have been considered desirable.

### Implications for practice

Medical incident reporting optimises improvement, deals with problems by implementing changes, improving awareness of the new and unforeseen issues requiring due attention, and creating a database of information. Thus, it provides evidence for further investigation and analysis, preventing future recurrence of similar incidents. Overall, it supports the healthcare environment in cultivating a safety culture requiring active and prompt involvement of all parties – including healthcare professionals, patients, and management.^19^

Therefore, it is essential to put forward and deliver good reports that are factual, well-detailed, and specific before the investigation takes place. This will help in the speedy recovery of the problems to support the investigation and mitigate the ongoing risks by devising accurate preventive and corrective strategies. We recommend the following measures for clinical practice based on the evidence obtained from this study and the considerations emerging from various literature and public reports. We believe these strategies will help healthcare professionals, analysts, relevant stakeholders, and organisations to bridge the gap in reporting incidents before and after investigation.

#### Facilitate adequate training sessions for healthcare professionals

One of the barriers to the digital incident reporting system includes reporters’ lack of knowledge about reporting and managing incidents.^20,21^ This hurdle has also been supplemented by the inability to cope with the regular workflow of the healthcare organisation and the functional complexity of the incident reporting system.^5,21^ The study by Anna Hahre indicated the need for extremely extensive training immediately after implementing the reporting system.^5^ The analysis suggested that the need for training sessions still existed even after 20 months of system implementation due to staff turnover. The instructional film to support the staff on how to report the incident was not found to be very useful because the healthcare organisation's workflow is time-constrained, and the healthcare professionals are always busy with their schedules.^5^

Moreover, the knowledge and competency required for the HIT system are at an advanced level in this area. Jabin et al. emphasised facilitating a training process as part of the professional development for the frontline operators with adequate paid time.^7^ The training sessions can also be supplemented by extra courses and education required for the reporters to align with the local, regional, and national standards of reporting.^22^ The ongoing refinement of professional development, training and education, and safeguards for the proper utilisation of the reporting systems should be accompanied by the existing endorsement of healthcare organisations’ quality practices. For instance, authentication and validation of user competency in reporting incidents should be introduced at the organisational level. The accreditation should signify the regular renewal of the operators’ credentials, specifically in case of workflow changes or the reporting system itself.^10^

As indicated by the findings of this study, training session should build a hands-on learning environment^23^ for the reporters to compare the manufacturer's conclusion for some of the incidents they have reported. This process would provide some context for the decision the reporter concluded and encourage the reporters to think outside of the proverbial box, leading to local quality improvement. The reporter's understanding of the manufacturer's conclusion would contribute to the better use and understanding of the HIT incidents that they may have to deal with on a regular basis.

#### Further work to agree on standard terms and definitions for the HIT systems

It is often impossible to devise preventive and corrective strategies for most of these technical problems due to inadequate or vague information in the incident reports. Due diligence is not paid to providing information by the reporters because they usually understand little of how HIT systems actually function. This problem is also accompanied by the lack of common terms and definitions used for HIT systems; therefore, different components of the systems and issues are generally regarded as ‘black boxes’. For example, the study by Jabin et al. specified that the term ‘error’ was used to indicate system malfunction or failures, and no difference was made between system interface or integration issues.^7^ These caused difficulties for the analysts in categorising the incidents; for instance, if standard definitions or terms had been available, some of the identified issues could have been assigned to other categories. Therefore, we propose further necessary work to agree on common definitions and preferred terms to frame standard HIT systems and the existing classification systems to bring cohesion to identifying and solving HIT-related issues.

#### Further work to refine the existing classification systems

Another prominent barrier to reporting incidents is the lack of consistency and validation of incident data classification.^21^ For example, a classification system in Australia was initiated focusing on the issues occurring in anaesthesiology but was further developed in the context of the general healthcare system.^24^ Later, due attention was paid to the full spectrum of healthcare proposed by the World Alliance for Patient Safety of the World Health Organization (WHO) to agree on common definitions and preferred terms to frame the International Classification for Patient Safety (ICPS).^25^ However, the ICPS was developed with the category of incident type ‘medical device’ without considering the HIT system.^17^ This necessitated a separate classification system, i.e., HIT-CS, exclusively required for classifying HIT-related incidents.^10^ Even though these classification systems complement each other, the contributing factors and the outcomes of the ICPS were found to be more comprehensive than those of HIT-CS.^17^ Therefore, there is a need for ongoing and iterative refinement of the existing classification systems in Sweden through research, continuous consultation, and advice-seeking with various healthcare stakeholders. The already existing classification should be refined and reinforced for incident categorisation, and healthcare professionals should be adequately trained before they are assigned the task of reporting incidents. Overall, there should be more detailed reporting before the incidents reach the investigation level, which will also prevent confusion between the roles at the reporting and investigation levels.

#### Enforce ‘mini’ root cause analysis at the reporting level

What follows is the narration of what and how a good incident reporting system should look like, based on the findings in this study and an appraisal of the literature. Incident reporting remains the only practical way to capture information about what goes wrong and why it goes wrong, particularly for rare events, to improve healthcare quality and safety.^26^ Such reporting systems should be online, non-punitive, accessible, valuable, and useable, and they may already exist in most jurisdictions.^27^ Ideally, the reporters should ensure the ‘cues’ used in the existing classification system, as discussed in the prior section. A structured ‘mini’ root cause analysis instead of an informal description should be included to ensure the completeness of the report. This should be supplemented by a mechanism for collecting further details from medicolegal files and complaints.^27^ This will lead to more detailed reporting of lesser incidents using the ‘cues’ and help orient the reporter and analysts to the tasks at each level, providing some context. It will also inform the analysts as to where preventive and corrective strategies should be put in place. Once each incident has been allotted to the appropriate step, the analyst may identify exactly what had happened and allow appropriate strategies to be determined. Therefore, a brief acknowledgement of receipt of the incident reports and continuous feedback on the analyses and the remedial measures are essential, ideally with a more system-wide approach.^17^

#### Establish a unit-based reporting system for the HIT incidents

Jabin,^17^ in his review of around 5000 incident reports using multiple classification systems, argued that any classification system is comprehensive on its own. For example, when such large data sets are analysed using thematic analysis, new themes and ideas emerge, which should ideally be added and incorporated into the existing classification systems, and incidents should then be coded according to the newly edited framework. Moreover, each healthcare department has its own type of problem. For example, the medical imaging department is more prone to HIT related challenges than others. The ICPS was not developed with consideration of the HIT system (rather ‘medical device’) in context, which necessitated a disparate framework, i.e., the HIT-CS for classifying HIT-related incidents.^17^

The Swedish MPA stipulated that incidents related to medical devices and national medical information systems should be reported to improve healthcare quality and mitigate the risks to patient safety.^6,28^ We ensure that a provision separating HIT incident reporting from medical devices is of utmost necessity due to their different salient features that would remove any confusion among healthcare professionals. For example, the regulation of HIT incidents (mostly focused on the National Health Service, mHealth, Telemedicine and Health data analytics) in the UK requires healthcare services to register under the Health and Social Care Act 2008, General Data Protection Regulation and the Data Protection Act 2018.

The authors suggest a unit-based reporting system since each healthcare department has its own type of problem^29^ at the local level. However, because the HIT systems are interconnected to each department, the provision of segregating HIT incidents should be made at the early stage. This will ensure a greater sense of urgency to devise locally applicable preventive and corrective strategies.

#### Ensure a standard incident reporting system at the national level

The incident reporting system in the UK has been centralised with the National Patient Safety Agency since 2003.^30^ However, in Sweden, there is a lack of a standard reporting system – the structure of healthcare incident reporting systems varies locally, regionally, and nationally. For example, the current Swedish incident reporting systems are decentralised, using different digital systems, such as Synergi, Platina, and LISA, in various regions.^5^ Therefore, these reporting systems are not liable to any accustomed healthcare quality standard, which paves the way to being under operational oversight of the system used in healthcare. This can be resolved by reinforcing the quality standard of the incident reporting systems at the national level; thus, viable management is the only way to overcome the challenges encountered at regional levels.

### Strengths and limitations of the study

A number of reasons have been assumed to be the cause of the low response rate. For example, this study was conducted during a busy and difficult moment for healthcare professionals, namely the coronavirus disease 2019 (COVID-19) pandemic. Additionally, a huge change in organisational structure and reorganisation at that moment restricted us from collecting more incidents as expected. However, incidents collected from the voluntary incident reporting system presented a sufficiently adequate holistic view of what went wrong in the routine Swedish healthcare practice concerning HIT systems with the narrative texts before and after the investigation. There were also a few drawbacks to the collected incident reports; for instance, two incidents had no narrative text provided (or it was missing), and the other two incidents had short narrative texts for the manufacturer's measures (after the investigation).

In the two years (2019–2021) for which the characteristics of the collected HIT incidents were involved from various regions, no transition of any emerging themes was observed. Therefore, measuring the effect of the HIT systems and their life cycle of implementation was not possible. There were other reasons for such inability to measure the impact: the decentralised nature of regional healthcare, the limited time period (2019–2021), and the small sample size.^18^ The sample, without a doubt, served the purpose of the research study to identify the barriers to incident reporting and how the context of the incidents changes after a thorough investigation of the reports by the analysts.

The consistency between the coders for classifying the incidents was performed using an inter-rater reliability test (kappa score calculation). Both primary and secondary coders underwent adequate experience in incident analysis and were previously engaged in examining an extensive data set of incident reports. Nevertheless, due attention should be paid to the findings since all the incidents were not representative of the category of patient safety events, particularly for the narrative texts described after the investigation.

A classification system, such as the HIT-CS, helps to examine incidents and draw out meaningful information, particularly for the classes – types of issues and consequences.^16^ Additional coding to check the involvement of either human or technical factors contributing to those incidents provided more insights and a measure of internal validation. For example, most of the use-related issues identified humans as contributing factors, whereas the most technical aspects caused the machine or software-related issues. This phenomenon ensures the recursive nature of incidents and the recurring association between types of issues and contributing factors (human versus technical). Hence, according to the phenomenon, the incident may contain a similar issue, a contributing factor, or both, based on the incident's salient features in that particular context.

Many studies have been published concerning the barriers or challenges to incident reporting systems, which are common in the USA,^31^ the UK,^32,33^ Australia,^21^ and European countries, such as Switzerland.^34^ Sweden is no exception in being susceptible to these problems; however, limited action has been taken to minimise these challenges.^35–[Bibr bibr36-20552076231174307]^^37^ Therefore, we suggest that the lessons learnt in this article can be conveniently practical and applicable elsewhere for studies related to healthcare quality improvement and patient safety.

## Conclusion

This study shed some light on the issues of incident reporting and the gap between the reporting and investigation levels. In conclusion, bridging the gap and minimising the challenges encountered at the reporting level to further develop the tools for reporting and managing incidents is worthy of further work. Facilitating sufficient training sessions for the staff, agreeing on common terms and definitions of the HIT systems, refining the existing classifications systems, enforcing mini-root cause analysis, and ensuring unit-based reporting at the local level and standard reporting at the national level may help overcome the challenges and bridge the gap between reporting and investigation levels in incident reporting.

## References

[1] Runciman WB Edmonds MJ Pradhan M . Setting priorities for patient safety. Qual Saf Health Care 2002; 11: 224–229. Research Support, Non-U.S. Gov’t 2002/12/19. 12486985 10.1136/qhc.11.3.224 PMC1743639

[2] Mandel C Runciman W . System for reporting and analysing incidents. In Radiological safety and quality: paradigms in leadership and innovation. Netherlands: Springer, 2014, pp. 203–221.

[3] Braithwaite J Wears RL Hollnagel E . Resilient health care: turning patient safety on its head. Int J Qual Health Care 2015; 27: 418–420. 26294709 10.1093/intqhc/mzv063

[4] Pronovost PJ Morlock LL Sexton JB , et al.Improving the value of patient safety reporting systems. In: Henriksen K Battles JB Keyes MA , et al. (eds) Advances in Patient Safety: New Directions and Alternative Approaches (Vol 1: Assessment). Rockville (MD): Agency for Healthcare Research and Quality, 2008. 21249859

[5] Hahre A . Usability in Incident Reporting Systems within health care organizations – A study on general perceptions and challenges. Malmö: Malmö University, 2016.

[6] Läkemedelsverket (Swedish Medical Product Agency). Incidents – medical devices, https://www.lakemedelsverket.se/en/reporting-adverse-reactions-events-and-incidents/incidents–medical-devices (accessed 28th January 2021).

[7] Jabin MSR Magrabi F Hibbert P , et al. Identifying and characterizing system issues of health information technology in medical imaging as a basis for recommendations. In: 2019 IEEE International Conference on Imaging Systems and Techniques (IST), 2019, pp. 1–6. Abu Dhabi: IEEE Xplore, DOI: 10.1109/IST48021.2019.9010426.

[8] Meeks DW Smith MW Taylor L , et al. An analysis of electronic health record-related patient safety concerns. J Am Med Inform Assoc 2014; 21: 1053–1059. 24951796 10.1136/amiajnl-2013-002578 PMC4215044

[9] Ash JS Berg M Coiera E . Some unintended consequences of information technology in health care: the nature of patient care information system-related errors. J Am Med Inform Assoc 2004; 11: 104–112. 14633936 10.1197/jamia.M1471 PMC353015

[10] Magrabi F Baker M Sinha I , et al. Clinical safety of England’s national programme for IT: a retrospective analysis of all reported safety events 2005 to 2011. Int J Med Inform 2015; 84: 198–206. 25617015 10.1016/j.ijmedinf.2014.12.003

[11] Castro GM Buczkowski L Hafner JM . The contribution of sociotechnical factors to health information technology-related sentinel events. Jt Comm J Qual Patient Saf 2016; 42: 70–76. 26803035 10.1016/s1553-7250(16)42008-8

[12] Basch E . New frontiers in patient-reported outcomes: adverse event reporting, comparative effectiveness, and quality assessment. Annu Rev Med 2014; 65: 307–317. 24274179 10.1146/annurev-med-010713-141500

[13] Walsh K Antony J . An assessment of quality costs within electronic adverse incident reporting and recording systems. Int J Health Care Qual Assur 2009; 22: 203–220. 19537183 10.1108/09526860910953494

[14] Subhedar NV Parry HA . Critical incident reporting in neonatal practice. Arch Dis Child Fetal Neonatal Ed 2010; 95: F378–F382. 19531520 10.1136/adc.2008.137869

[15] Buckley TA Short TG Rowbottom YM , et al. Critical incident reporting in the intensive care unit. Anaesthesia 1997; 52: 403–409. 9165956 10.1111/j.1365-2044.1997.094-az0085.x

[16] Jabin MSR Magrabi F Hibbert P , et al. Identifying and classifying incidents related to health information technology in medical imaging as a basis for improvements in practice. In: *2019 IEEE International Conference on Imaging Systems and Techniques (IST)*, 2019, pp. 1–6. Abu Dhabi: IEEE Xplore, DOI: 10.1109/IST48021.2019.9010109.

[17] Jabin MSR . Identifying and characterising problems arising from interactions between medical imaging and health information technology as a basis for improvements in practice. Adelaide: University of South Australia, 2019.

[18] Jabin MSR Pan D Nilsson E . Characterizing healthcare incidents in Sweden related to health information technology affecting care management of multiple patients. Health Inform J 2022; 28, DOI: 14604582221105440. 10.1177/14604582221105440 35762538

[19] Pham JC Girard T Pronovost PJ . What to do with healthcare incident reporting systems. J Public Health Res 2013; 2: e27. 25170498 10.4081/jphr.2013.e27 PMC4147750

[20] Lee W Kim SY Lee SI , et al. Barriers to reporting of patient safety incidents in tertiary hospitals: a qualitative study of nurses and resident physicians in South Korea. Int J Health Plann Manage 2018; 33: 1178–1188. 30160794 10.1002/hpm.2616

[21] Evans SM Berry JG Smith BJ , et al. Attitudes and barriers to incident reporting: a collaborative hospital study. Qual Saf Health Care 2006; 15: 39–43. 16456208 10.1136/qshc.2004.012559 PMC2563993

[22] Saner H van der Velde E . Ehealth in cardiovascular medicine: a clinical update. Eur J Prev Cardiol 2016; 23: 5–12. 27892420 10.1177/2047487316670256

[23] Lewis AM Sordo S Weireter LJ , et al. Mass casualty incident management preparedness: a survey of the American college of surgeons committee on trauma. Am Surg 2016; 82: 1227–1231. 28234189

[24] Runciman WB Helps SC Sexton EJ , et al. A classification for incidents and accidents in the health-care system. J Qual Clin Pract 1998; 18: 199–211. Research Support, Non-U.S. Gov’t 1998/09/23. 9744659

[25] Runciman WB Hibbert P Thomson R , et al. Towards an International Classification For Patient Safety: key concepts and terms. Int J Qual Health Care 2009; 21: 18–26. Research Support, Non-U.S. Gov’t 2009/01/17. 19147597 10.1093/intqhc/mzn057 PMC2638755

[26] Kinnunen UM Sarantob K . It is time for self-incident-reporting for patients and their families in every health care organization: a literature review. Stud Health Technol Inform 2013; 192: 92–96. 23920522

[27] Runciman WB Williamson JA Deakin A , et al. An integrated framework for safety, quality and risk management: an information and incident management system based on a universal patient safety classification. Qual Saf Health Care 2006; 15: i82–i90. Review 2006/12/05. 17142615 10.1136/qshc.2005.017467 PMC2464872

[28] Nygren M Roback K Öhrn A , et al. Factors influencing patient safety in Sweden: perceptions of patient safety officers in the county councils. BMC Health Serv Res 2013; 13: 52. 23391301 10.1186/1472-6963-13-52 PMC3579677

[29] Wagner C Merten H Zwaan L , et al. Unit-based incident reporting and root cause analysis: variation at three hospital unit types. BMJ Open 2016; 6: e011277. 10.1136/bmjopen-2016-011277 PMC4916568 27329443

[30] Hutchinson A Young TA Cooper KL , et al. Trends in healthcare incident reporting and relationship to safety and quality data in acute hospitals: results from the national reporting and learning system. Qual Saf Health Care 2009; 18: 5–10. 19204125 10.1136/qshc.2007.022400

[31] Schectman JM Plews-Ogan ML. Physician perception of hospital safety and barriers to incident reporting. Jt Comm J Qual Patient Saf 2006; 32: 337–343. 16776388 10.1016/s1553-7250(06)32043-0

[32] Lawton R Parker D . Barriers to incident reporting in a healthcare system. Qual Saf Health Care 2002; 11: 15. 12078362 10.1136/qhc.11.1.15 PMC1743585

[33] Firth-Cozens J . Barriers to incident reporting. Qual Saf Health Care 2002; 11: 7. 10.1136/qhc.11.1.7 PMC1743555 12120590

[34] Pfeiffer Y Manser T Wehner T . Conceptualising barriers to incident reporting: a psychological framework. Qual Saf Health Care 2010; 19: e60. 20558472 10.1136/qshc.2008.030445

[35] Carlfjord S Öhrn A Gunnarsson A . Experiences from ten years of incident reporting in health care: a qualitative study among department managers and coordinators. BMC Health Serv Res 2018; 18: 113. 29444680 10.1186/s12913-018-2876-5 PMC5813432

[bibr36-20552076231174307] Björne P Deveau R Nylander L . Passing laws is not enough to change staff practice: the case of legally mandated “incident” reporting in Sweden. J Intellect Dev Disabil 2021; 46: 186–196. 10.3109/13668250.2021.1873751 39818545

[37] Jabin MSR Hammar T . Issues with the Swedish e-prescribing system – an analysis of health information technology-related incident reports using an existing classification system. Digital Health Journal 2022. DOI: 10.1177/20552076221131139. PMC9554230 36249479

